# Clinical and genetic profiles of patients with hereditary and wild-type transthyretin amyloidosis: the Transthyretin Cardiac Amyloidosis Registry in the state of São Paulo, Brazil (REACT-SP)

**DOI:** 10.1186/s13023-024-03281-z

**Published:** 2024-07-20

**Authors:** Fábio Fernandes, Georgina del Cisne Jadán Luzuriaga, Guilherme Wesley Peixoto da Fonseca, Edileide Barros Correia, Alzira Alves Siqueira Carvalho, Ariane Vieira Scarlatelli Macedo, Otavio Rizzi Coelho-Filho, Phillip Scheinberg, Murillo Oliveira Antunes, Pedro Vellosa Schwartzmann, Sandrigo Mangini, Wilson Marques, Marcus Vinicius Simões

**Affiliations:** 1 Academic Research Organization, Instituto do Coração InCor, Hospital das Clínicas HCFMUSP, Faculdade de Medicina, Universidade de São Paulo, São Paulo, Brazil; 2 Instituto do Coração InCor, Hospital das Clínicas HCFMUSP, Faculdade de Medicina, Universidade de São Paulo, São Paulo, Brazil; 3 Instituto Dante Pazzanese de Cardiologia, São Paulo, Brazil; 4 Centro Universitário de Saúde ABC, Santo André, Brazil; 5 Instituto de Pesquisa E Inovação Tecnológica da Santa Casa de São Paulo, São Paulo, Brazil; 6 School of Medical Science, University of Campinas, UNICAMP, Campinas, Brazil; 7 Beneficência Portuguesa de São Paulo, São Paulo, Brazil; 8 Hospital Universitário São Francisco na Providência de Deus, Bragança Paulista, Brazil; 9 CLINICOR Clínica Cardiológica Ltda., Ribeirão Preto, Brazil; 10 Hospital Israelita Albert Einstein, São Paulo, Brazil; 11 Hospital das Clínicas da Faculdade de Medicina de Ribeirão Preto da Universidade de São Paulo, Ribeirão Preto, Brazil

**Keywords:** Cardiac Amyloidosis, Epidemiology, Polyneuropathy, Registry, Transthyretin Amyloidosis

## Abstract

**Background:**

Transthyretin amyloidosis (ATTR) is a multisystem disease caused by the deposition of fibrillar protein in organs and tissues. ATTR genotypes and phenotypes are highly heterogeneous. We present data on physical signs and symptoms, cardiac and neurological assessments and genetic profile of patients enrolled in the Transthyretin Cardiac Amyloidosis Registry of the State of São Paulo, Brazil.

**Results:**

Six hundred-forty-four patients were enrolled, 505 with the variant form (ATTRv) and 139 with wild-type (ATTRwt). Eleven different mutations were detected, the most common being Val50Met (47.5%) and V142Ile (39.2%). Overall, more than half of the patients presented cardiac involvement, and the difference in this proportion between the ATTRv and ATTRwt groups was significant (43.9 vs. 89.9%; *p* < 0.001). The prevalence of the neurological phenotype also differed between ATTRv and ATTRwt (56.8 vs. 31.7%; *p* < 0.001). The mixed phenotype was found in 25.6% of the population, without a significant difference between ATTRv and ATTRwt groups. A group of patients remained asymptomatic (10.4%), with a lower proportion of asymptomatic ATTRwt patients.

**Conclusions:**

This study details the clinical and genetic spectrum of patients with ATTR in São Paulo, Brazil. This preliminary analysis highlights the considerable phenotypic heterogeneity of neurological and cardiac manifestations in patients with variant and wild-type ATTR.

## Background

Transthyretin amyloidosis (ATTR) is a multisystem disease caused by the deposition of fibrillar protein in organs and tissues. This condition is being diagnosed more frequently despite being considered a rare disease [1]. The disorder develops due to the extracellular deposition of misfolded transthyretin (TTR) protein in the form of amyloid fibrils in tissues. This process involves several mechanisms and begins when the tetrameric form of unstable TTR dissociates into dimers and monomers [2]. Pathophysiological hypotheses about the mechanisms that cause TTR to become unstable include genetic variants, in which case the disease is called the variant (ATTRv) form, or processes involving oxidative changes in the protein secondary to aging, in which case the disease is named the wild-type (ATTRwt) form.

The clinical presentation varies widely, from asymptomatic to severe cardiological and/or neurological involvement. Recently, significant advances in knowledge about ATTR have been made, leading to greater recognition of its clinical picture and epidemiology with increasing treatment possibilities. Despite being an incurable disease, new treatments are associated with slowing the progression of ATTR with cardiomyopathy (ATTR-CM) and polyneuropathy (ATTR-PN). Greater awareness about the disease among general physicians and specialists has contributed to reducing the delay in diagnosis and the rate of misdiagnosis [1].

ATTR-PN usually manifests as progressive sensory, motor and autonomic polyneuropathy [3]. In ATTR-CM, myocardial interstitial deposition of TTR fibrillar aggregates can cause functional and anatomical cytotoxicity in cardiomyocytes [4]. ATTR-CM is an under-recognized, progressive, and often fatal disease. In the early phase of the disease, the symptoms may mimic hypertensive heart disease, hypertrophic cardiomyopathy, and heart failure (HF) with preserved ejection fraction, atrial and ventricular arrhythmias, and aortic and mitral disease [5–7]. Untreated, the disease relentlessly progresses, causing severe disability and ultimately death.

Patients affected by amyloidosis require screening, diagnosis, monitoring, and specific therapeutic strategies. In Brazil, the available data regarding the prevalence of ATTR, both ATTRwt and ATTRv, are scarce and remain mostly unexplored.

We designed the Transthyretin Cardiac Amyloidosis Registry in the state of São Paulo (REACT-SP), aiming to characterize the demographic, genetic, clinical, diagnostic test results and undergoing treatment of patients with ATTR.

## Methods

REACT-SP is an observational and multicenter registry study of patients diagnosed with ATTR. The HCFMUSP Ethics Committee approved this study, and written informed consent was waived. (CAAE: 53,302,721.5.1001.0068; Approval report No. 5.188.324). The data were retrospectively collected from February 11, 2022 until July 3, 2023.

Ten centers participated: Instituto do Coração- InCor, Hospital das Clínicas HCFMUSP, Faculdade de Medicina, Universidade de São Paulo (coordinating center), Instituto Dante Pazzanese de Cardiologia, Hospital Israelita Albert Einstein, Hospital das Clínicas, Faculdade de Medicina de Ribeirão Preto, Universidade de São Paulo, CLINICOR Clínica Cardiológica Ltda., Hospital das Clínicas, Universidade Estadual de Campinas UNICAMP, Hospital Beneficência Portuguesa de São Paulo, Hospital Universitário São Francisco na Providência de Deus, Centro Universitário de Saúde ABC and Instituto de Pesquisa e Inovação Tecnológica, Santa Casa de São Paulo.

Information regarding demographic, genetic, clinical, imaging and treatment methods was extracted from the patients' medical records at the originating services after the last follow-up consultation and entered into the online platform Research Electronic Data Capture (REDCap).

Patients aged > 18 years and diagnosed with ATTRv or ATTRwt with a cardiologic, neurologic or mixed phenotype were included. Patients with other forms of amyloidosis (such as AL amyloidosis, secondary or localized amyloidosis) were excluded.

Comorbidities were organized into hypertension, diabetes mellitus, dyslipidemia, coronary artery disease, chronic kidney disease (CKD, defined by a glomerular filtration rate < 60 mL/min/1.73m^2^ more than three months) and carpal tunnel syndrome. The clinical parameters evaluated were body mass index (BMI), heart rate, systolic and diastolic blood pressure and oxygen saturation in room air. The medical specialties that made the diagnosis of amyloidosis were cardiology, neurology, hematology, oncology, psychiatry and others. The initial clinical manifestations were categorized as asymptomatic, paresthesia or loss of strength in the extremities, dyspnea, diarrhea, arrhythmia, dysautonomia and stroke. The laboratory values, hemoglobin, C-reactive protein, cardiac biomarkers (NTpro-BNP and troponin-I), renal function (urea and creatinine), albumin and fasting blood glucose were evaluated. The functional capacity was also described according to the New York Heart Association functional class (NYHA-FC) I-IV.

Cardiac involvement was defined by the presence of a mean thickness of the left ventricular wall > 12 mm by echocardiography and the presence of at least one of the following parameters: TTR amyloid in cardiac tissue confirmed by mass spectrometry analysis, immunohistochemistry, or cardiac scintigraphy with the bone tracer ^99m^technetium-pyrophosphate (^99m^Tc-PYP) with an uptake level ≥ 2 on the Perugini scale. In all cases, primary amyloidosis was ruled out by light chain kappa and lambda immunoglobulin measurement and serum and urinary immunofixation. Neurologic involvement was defined by the presence of clinical sensorimotor manifestations such as paresthesia, loss of strength in the extremities, autonomic dysfunction (orthostatic hypotension, gastrointestinal disorders or neurogenic bladder), stroke or carpal tunnel syndrome. We also collected data on continuous treatment with cardiologic medications and amyloidosis-specific therapies.

Imaging findings from electrocardiography (ECG), 24-h Holter monitoring, echocardiography, cardiac scintigraphy with ^99m^Tc-PYP, and cardiac magnetic resonance (CMR) were evaluated when available. The ECG parameters included sinus rhythm, atrial fibrillation/flutter (AF), low QRS voltage, ventricular repolarization disturbances and the presence of an inactive area. The 24-h Holter analysis included mean heart rate (HRm) and density of ventricular and supraventricular extrasystoles (as percentages and per hour).

The echocardiogram results were left ventricular ejection fraction (LVEF), interventricular septal thickness (IVS), posterior wall thickness (PWT), left atrium (LA) diameter, left ventricular diastolic and systolic diameter (LVDD and LVSD), basal right ventricle diastolic diameter (RVDD), presence of apical sparing (AS) by global analysis of longitudinal myocardial deformation, LV diastolic dysfunction and presence of thrombus or intracavitary masses. The AS value was manually calculated as the ratio of Longitudinal strain in apical to non-apical (basal and mid) LV segments, based on a 16-segment LV model. The ^99m^Tc-PYP scintigraphy was evaluated at 1 h and 3 h after tracer administration. CMR was used to analyze T1 map values and extracellular volume (ECV) and to assess late gadolinium enhancement (LGE). If available, the tissue biopsy sites for diagnosis were categorized into gastrointestinal tract, heart, skin, nervous tissue and others.

### Statistical analysis

Continuous variables are presented as mean ± standard deviation or median with interquartile range (IQR), and categorical variables are presented as frequencies and percentages. One sample Kolmogorov–Smirnov test was applied to assess the normality of the distribution of the studied variables. Independent Student’s t tests and Mann–Whitney U tests were used to compare parametric and nonparametric variables, respectively. The chi-square test (χ2 test) was used to compare differences between categorical variables. The data were analyzed using the Statistical Package for the Social Sciences Version 23 for Windows (SPSS, Inc., Chicago, IL, USA). A p value lower than 0.05 was considered statistically significant for all analyses.

## Results

### Patient demographic characteristics

We included 644 patients, predominantly male (62.8%), with a median age of 65 years. Regarding race, the majority were white (74.5%). In comparison to wild type, the ATTRv patients significantly differed regarding ethnicity (white race in 74.5% vs. 86.5%; *p* = 0.002), sex (male in 59.4% vs. 75.5%; *p* < 0.001) and age (54 vs. 78 years; *p* < 0.001). The most frequent comorbidities were hypertension (26.9%), dyslipidemia (15.1%), carpal tunnel syndrome (13.0%), and chronic kidney disease (13.0%), with a higher incidence of comorbidities occurring in ATTRwt patients (Table 1).

**Table 1:** Clinical and demographic characteristics for all patients and for amyloidosis type

	All patients (*n* = 644)	ATTRv (*n* = 505)	ATTRwt (*n* = 139)	*p* value
Age, median years	65 (46–75)	54 (43–70)	78 (71–84)	< 0.001
Male, n (%)	405 (62.8)	300 (59.4)	105 (75.5)	0.001
*Ethnicity, n (%)*	*n* = 609	*n* = 483	*n* = 126	
White	454 (74.5)	345 (71.4)	109 (86.5)	0.002
Black	153 (25.1)	137 (28.4)	16 (12.7)	0.002
*Comorbidities, n (%)*				
Hypertension	173 (26.9)	113 (22.4)	60 (43.2)	< 0.001
Diabetes Mellitus	61 (9.5)	35 (6.9)	26 (18.7)	< 0.001
Dyslipidemia	97 (15.1)	64 (12.7)	33 (23.7)	< 0.001
Coronary artery disease	25 (3.9)	11 (2.2)	14 (10.1)	< 0.001
Chronic renal disease	84 (13.0)	54 (10.7)	30 (21.6)	< 0.001
Carpal tunnel syndrome	88 (13.7)	61 (12.1)	27 (19.4)	0.020
*Clinical parameters, median*				
BMI, kg/m^2^	24.2 (21.5–27.5)	24.0 (21.0–27.3)	25.7 (22.7–28.2)	0.006
Heart rate, bpm	74 (67–83)	73 (66–84)	75 (68–82)	0.521
SBP, mmHg	120 (106–132)	120 (106–131)	120 (109–138)	0.064
DBP, mmHg	73 (65–80)	74 (66–82)	73 (65–82)	0.913
Saturation O_2_, %	97 (96–98)	97 (96–98)	97 (95–98)	0.346
*Medical specialty that diagnosed, n (%)*				
Cardiology	263 (40.8)	168 (33.3)	95 (68.3)	< 0.001
Neurology	91 (14.1)	88 (17.4)	3 (2.2)	< 0.001
Hematology	9 (1.4)	6 (1.2)	3 (2.2)	0.412
Oncology	4 (0.6)	2 (0.4)	2 (1.4)	0.202
Psychiatry	4 (0.6)	4 (0.8)	0 (0.0)	0.583
Others^a^	89 (13.8)	85 (16.8)	4 (2.9)	< 0.001
*Initial symptom, median days*	1853 (1277–2997)	2013 (1448–3109)	1648 (1047–2378)	< 0.001
*First symptom, n (%)*				
Asymptomatic	67 (10.4)	64 (12.6)	3 (2.1)	< 0.001
Paresthesias of extremities	198 (30.7)	184 (36.4)	14 (10.1)	< 0.001
Dyspnea	165 (25.5)	101 (20.0)	64 (46.0)	< 0.001
Diarrhea	34 (5.3)	32 (6.3)	2 (1.4)	0.018
Arrhythmia	31 (4.8)	16 (3.2)	15 (10.8)	< 0.001
Dysautonomia	22 (3.4)	18 (3.6)	4 (2.9)	1.000
Stroke	8 (1.2)	4 (0.8)	4 (2.9)	0.069
*Laboratory parameters, median*				
Hemoglobin, g/dL	13.9 (12.4–14.9)	13.8 (12.4–14.9)	14.0 (12.4–15.0)	0.725
R-reactive protein, mg/L	3.3 (1.3–12.0)	3.0 (1.0–11.7)	5.8 (1.8–13.0)	0.256
BNP, pg/mL	161.0 (48.0–538.0)	92.0 (310.3–855.3)	524.0 (310.3–855.3)	< 0.001
NTpro-BNP, pmol/L	813.0 (85.3–3066.5)	429.0 (53.0–213.0)	1764.5 (1006.3–4364.3)	< 0.001
Troponin-I, ng/mL	10.0 (3.0–39.8)	9.0 (3.0–34.5)	29.0 (0.5–67.0)	0.176
Urea, mg/dL	45.5 (34.0–64.8)	41.0 (30.0–58.0)	60.0 (46.0–87.0)	< 0.001
Creatinine, mg/dL	1.1 (0.9–1.5)	1.0 (0.8–1.3)	1.4 (1.1–1.8)	< 0.001
Albumin, mmol/L	4.2 (3.7–4.5)	4.2 (3.8–34.5)	4.0 (3.6–4.3)	0.670
Fasting glucose, mg/dL	93 (85–107)	93 (85–106)	98 (87–110)	0.229
*Phenotype, n (%)*				
Cardiologic	347 (53.9)	222 (43.9)	125 (89.9)	< 0.001
Neurologic	331 (51.3)	287 (56.8)	44 (31.7)	< 0.001
Mixed	165 (25.6)	130 (25.7)	35 (25.2)	1.000
*NYHA functional class, n (%)*	*n* = *241*	*n* = *162*	*n* = *79*	
I	38 (15.8)	27 (16.7)	11 (13.9)	0.380
II	137 (56.8)	92 (56.8)	45 (57.0)	
III	51 (21.2)	35 (21.6)	16 (20.3)	
IV	15 (6.2)	8 (4.9)	7 (8.9)	
*Medication, n (%)*				
Diuretics	215 (33.4)	128 (25.3)	87 (62.6)	< 0.001
ACEIs/ARBs	107 (16.6)	71 (14.1)	36 (25.9)	0.002
ARNIs	38 (5.9)	21 (4.2)	17 (12.2)	0.001
SGLT2-Is	75 (11.6)	38 (7.5)	37 (26.6)	< 0.001
Beta-blockers	133 (20.7)	72 (14.3)	61 (43.9)	< 0.001
Mineralocorticoid receptor antagonist	130 (20.2)	81 (16.0)	49 (35.3)	< 0.001
Antiarrhythmic	70 (10.9)	37 (7.3)	33 (23.7)	< 0.001
Anticoagulant	34 (5.3)	22 (4.4)	12 (8.6)	0.054
*Specific drug therapies in ATTR, n (%)*				
Tafamidis	110 (17.1)	95 (18.8)	15 (10.8)	0.030
Doxycycline	8 (1.2)	4 (0.8)	4 (2.9)	0.069
Patisiran	12 (1.9)	8 (1.6)	4 (2.9)	0.970
Inotersen	12 (1.9)	9 (1.8)	3 (2.2)	0.726
AG10	2 (0.3)	0 (0.0)	2 (1.4)	0.046
Other^b^	1 (0.2)	0 (0.0)	1 (0.7)	0.214

Continuous variables are presented as median with interquartile range and categorical variables as n (%)

*ChBMI* Body mass index, *SBP* Systolic blood pressure, *DBP* Diastolic blood pressure, *BNP* B-type natriuretic peptide, *NTpro-BNP* N-terminal pro–B-type natriuretic peptide, *NYHA* New York Heart Association, *ACEIs/ARBs* Angiotensin-converting-enzyme inhibitors/angiotensin receptor blockers, *ARNIs* Angiotensin receptor/neprilysin inhibitors, *SGLT2-Is* Sodium-glucose cotransporter-2 inhibitors, *ATTR* Transthyretin amyloidosis

^a^Gastroenterology, orthopedic

^b^Green tea

### Genetic characteristics

Eleven variants in the TTR gene were reported, the most frequent being Val50Met (47.5%) and Val142Ile (39.2%) (Table 2). Other variants in the TTR gene were reported: Thr80Ala 2.37%, Ala39Asp 1.38%, Phe64Ser 1.18%, Glu109Lys 1.18% and additional ones.

**Table 2:** Detected mutations in the transthyretin gene

Genes	Values (%)
Val50Met	47.5
Val142Ile	39.2
Thr80Ala	2.37
Ala39Asp	1.38
Phe64Ser	1.18
Glu109Lys	1.18
Gly67Glu	0.99
Phe84Leu	0.59
Others	5.54

Genes Variants in the TTR are presented as categorical variables as n (%)

### Clinical and imaging characteristics and treatment

Regarding clinical parameters, the median BMI was 24.2 (IQR 21.5–27.5) kg/m^2^, and there was a significant difference between the ATTRv and ATTRwt groups (24.0 vs. 25.7%; *p* = 0.006). Median heart rate was 74 (IQR 67–83) bpm. Median systolic blood pressure was 120 (IQR 106–132) mmHg, and median diastolic blood pressure was 73 (IQR 65–80) mmHg; there was no difference between the groups.

The specialties that most frequently diagnosed ATTR were cardiology (40.8%), neurology (14.1%) and hematology (1.4%). The median time between the onset of symptoms and diagnosis was 1,853 (IQR 1277–2997) days, which was longer in ATTRv patients than in ATTRwt patients (*p* < 0.001). The most common first symptoms reported were paresthesia in the extremities (30.7%), dyspnea (25.5%), diarrhea (5.3%), arrhythmia (4.8%), dysautonomia (3.4%) and stroke (1.2%), although a group of patients remained asymptomatic (10.4%), with a lower proportion of asymptomatic patients among the ATTRwt patients. Symptoms related to polyneuropathy were more frequent in ATTRv patients, while symptoms related to heart disease (dyspnea and arrhythmia) were more frequent in ATTRwt patients. Dysautonomic manifestations were not different between the ATTRv and ATTRwt groups. Regarding biochemical blood tests, the median BNP was 161.0 pg/mL, and the median NTpro-BNP was 813.0 pmol/L, with higher values observed in ATTRwt patients (*p* < 0.001). Troponin-I averaged 10 ng/mL, with no difference between groups (*p* = 0.176).

More than half of the patients presented cardiac involvement, and the difference between the ATTRv and ATTRwt groups was significant (43.9 vs. 89.9%; *p* < 0.001). In parallel, the neurological phenotype also differed between ATTRv and ATTRwt (56.8 vs. 31.7%; *p* < 0.001). The mixed phenotype was found in 25.6% of the population, without a significant difference between the forms of amyloidosis (*p* = 1.000). The majority of patients were classified as NYHA-FC II (56.8%). Among the specific drug therapies in ATTR, the most frequently reported was tafamidis, accounting for 17.1% of cases. The others medications were done by participation of the patients in multicenter studies (Table 1).

An ECG was available for 329 patients. Sinus rhythm was reported in 74.2%, AF in 17.0%, low QRS voltage in 28.6%, repolarization abnormalities in 39.8% and electrical inactive area in 27.4%. Among these characteristics, sinus rhythm was more frequent in ATTRv patients than in ATTRwt patients, while AF was more frequent in ATTRwt patients. Twenty-four-hour Holter monitoring was performed for 155 patients, revealing a lower HRm in ATTRwt patients and higher density of ventricular extrasystoles/hours than in ATTRv patients (Table 3).

**Table 3:** Cardiovascular data for all patients and for the two disease types

	All patients	ATTRv	ATTRwt	*p* value
*Electrocardiogram*	(*n* = 329)	(*n* = 250)	(*n* = 79)	
Sinus Rhythm, n (%)	244 (74.2)	203 (81.2)	41 (51.9)	0.049
Atrial fibrillation/Flutter, n (%)	56 (17.0)	27 (10.8)	29 (36.7)	< 0.001
Low voltage, n (%)	94 (28.6)	70 (28.0)	24 (30.4)	0.774
Repolarization abnormalities, n (%)	131 (39.8)	94 (37.6)	37 (46.8)	0.103
Inactive area, n (%)	90 (27.4)	64 (25.6)	26 (32.9)	0.186
*24-h Holter, median*	(*n* = 155)	(*n* = 123)	(*n* = 32)	
Mean heart rate	74 (66–84)	78 (68–85)	68 (63–77)	0.004
EEVV density, %	1.0 (1.0–1.0)	3.0 (0.2–30.0)	1.0 (1.0–1.0)	0.868
EEVV/h	4.0 (0.3–30.5)	77.5 (65.0–85.0)	15.5 (2.0–46.5)	0.025
ESV density, %	1.0 (1.0–1.0)	2.0 (0.4–21.0)	1.0 (1.0–1.0)	0.570
ESV/h	5.0 (0.5–34.4)	1.0 (1.0–1.0)	25.0 (8.8–336.3)	0.020
*Echocardiogram, median*	(*n* = 383)	(*n* = 291)	(*n* = 92)	
LVEF, %	60 (49–65)	62 (54–67)	53 (43–60)	< 0.001
IVS, mm	14 (10–17)	13 (9–16)	15 (13–17)	< 0.001
PWT, mm	13 (9–15)	12 (9–15)	14 (13–16)	< 0.001
LA, mm	41 (34–47)	38 (32–45)	46 (42–51)	< 0.001
LVDD, mm	45 (41–48)	45 (41–47)	46 (42–49)	0.049
LVSD mm	30 (27–35)	29 (27–34)	33 (30–37)	< 0.001
RVDD, mm	35 (30–39)	35 (28–39)	37 (31–40)	0.114
LV longitudinal strain, %	9.1 (7.1–15.3)	10.9 (6.9–16.9)	10.3 (7.9–12.4)	0.272
LV diastolic dysfunction, n (%)	147 (38.4)	101 (34.7)	46 (50.0)	< 0.001
Apical Sparing, n (%)	120 (31.3)	82 (28.2)	38 (42.2)	0.035
Thrombi or masses, n (%)	4 (1.0)	2 (0.7)	2 (2.2)	0.234
^*99m*^*Tc-PYP scintigraphy, median*	(*n* = 349)	(*n* = 260)	(*n* = 89)	
1-h	1.7 (1.4–1.9)	1.6 (1.2–1.8)	1.8 (1.6–1.9)	< 0.001
3-h	1.5 (1.1–1.8)	1.3 (1.1–1.6)	1.7 (1.5–1.8)	< 0.001
*LGE, n (%)*	(*n* = 183)	(*n* = 138)	(*n* = 45)	
Absent	53 (29.0)	49 (35.3)	4 (8.9)	0.017
Subendocardial	65 (35.5)	39 (28.1)	26 (57.8)	< 0.001
Mesocardial	25 (13.7)	18 (12.9)	7 (15.6)	0.205
Transmural	25 (13.7)	20 (14.4)	6 (13.3)	0.624
Epicardial	15 (8.2)	13 (9.4)	2 (4.4)	0.746
*Cardiac magnetic resonance, median*	(*n* = 43)	(*n* = 32)	(*n* = 11)	
T1 map, ms	1067.0 (999.0–1165.0)	1090.0 (1004.6–1174.5)	1064.0 (958.0–1067.0)	0.037
ECV, %	47.5 (26.2–56.5)	38.5 (24.1–51.5)	55.1 (54.0–60.0)	0.002

Continuous variables are presented as median with interquartile range and categorical variables as n (%)

*LVEF* Left ventricle ejection function, *IVS* Interventricular septum, *PWT* Posterior wall thickness, *LA* Left atrium, *LVDD* Left ventricular diastolic diameter, *LVSD* Left ventricular systolic diameter, *RVDD* Right ventricle diastolic diameter, *LV* Left ventricle, *99mTc-PYP* 99 m-technetium-pyrophosphate, *LGE* Late gadolinium enhancement, *ECV* Extracellular volume

Echocardiography was available for 383 patients. The median LVEF was 60%, IVS 14 mm, PWT 13 mm, LA 41 mm, LVDD 45 mm, LVSD 30 mm, basal RVDD 35 mm and LV longitudinal strain 9.1%. There was some degree of LV diastolic dysfunction in 147 patients (38.4%), apical sparing in 120 patients (31.3%) and thrombus in 4 patients (1.0%). There were statistically significant differences between ATTR forms in all of these measures besides baseline RVDD, LV longitudinal strain, and the presence of thrombi or masses. Cardiac scintigraphy was available for 349 patients. Its values differed between groups at 1 h (1.6 vs. 1.8; *p* < 0.001) and 3 h (1.3 vs. 1.7; *p* < 0.001) after applying the radiopharmaceutical (Table 3).

Data were obtained from 183 patients with CMR. LGE was present in 130 patients (71%)and the patterns were subendocardial in 65 patients (35.5%), mesocardial in 25 patients (13.7%), transmural in 25 patients (13.7%) and epicardial in 15 patients (8.2%) (Table 3) Moreover, significant differences were observed in the T1 mapping data between ATTRv and ATTRwt patients. Those with ATTR, likely reflecting a later diagnosis with more pronounced cardiac involvement, were more likely to show higher T1 values and increased ECV compared to individuals with ATTRv (Table 3).

Biopsy was performed on 107 patients, the most frequent sites being the gastrointestinal tract (56.1%), heart (17.8%), skin (9.3%), nervous tissue (5.6%) and others (11.2%).

## Discussion

This is the largest registry in Brazil to describe the demographic, genetic, and clinical imaging characteristics and treatment of patients diagnosed with ATTR. We observed that there was a greater proportion of the variant form, contrasting with other studies [2, 6]. The most frequently associated mutation in this study was Val50Met, followed by Val142Ile. The high frequence of Val50Met in Brazil has already been reported and probably reflects relevant Portuguese participation in the ethnic composition of Brazilian population [8]. On the other hand, the high proportion of Val142Ile is concordant with previous results of studies investigating american populations with ethnic composition including afrodescendents [9]. There were also other mutations that have been reported in other areas of the world, such as Thr80Ala, Ala39Asp, Phe64Ser, Glu109Lys [10–15]. The variety of mutations found might be explained by the fact that São Paulo is the most populated state in Brazil, the largest country in South America, housing population groups from other regions of the globe.

The median age was 65 years, which is consistent with other studies that showed a higher incidence in the population > 63 years of age [6, 16–18]. We observed that patients with ATTRv were younger (median of 54 years of age) than patients with ATTRwt, in line with some studies [19], though others have reported a median age of 73 years in ATTRv patients [20]. This is possibly because these patients were from different geographic regions where the epigenetic factors to which the population is exposed differ.

Of the whole sample, 62.8% were male, which coincides with the findings of a systematic review that compared 4,669 patients with ATTR by sex, revealing a male predominance of 83% [21]. This could be due to low clinical suspicion, which reduces the likelihood of diagnosis in women, in addition to the likely cardioprotection caused by estrogens. Although this is a hypothesis, the study by Prasad et al. demonstrated that the identification of this disease in females has increased, with the percentage of women diagnosed with ATTR-CM increasing from 5.9% before 2019 to 16.7% from 2019 to 2022 [22].

In this study, the majority of patients were white (74.5%), as in the study by Porcari et al., in which there was a higher prevalence of amyloidosis in white patients (80.4%). This predilection is notable, but it could be due to the population base, as these patients were from the United Kingdom [20]. The most associated comorbidities were arterial hypertension, CKD and dyslipidemia, which were also associated with ATTR according to previous studies [20, 23–25].

We determined that among medical specialties, cardiologists most frequently diagnosed ATTR, a finding also obtained by other researchers [25]. In this study, this could have been because the centers mostly specialized in cardiology.

The time from the onset of symptoms to the diagnosis of amyloidosis is essential, as it has been directly related to survival in patients [25]. In this study, the duration of disease before diagnosis was still high with 1853 days, which is in agreement with previous studies [9, 25]. However, the awareness of the disease in the last few years has dramatically increased that could impact in the future in an early diagnosis for the patients.

The initial symptoms were neurological and were mainly referred to as paresthesia in the extremities and cardiological symptoms, such as dyspnea. This clinical heterogeneity was due to the systemic involvement of the disease.

In this study, the neurological phenotype was present in 51.1% of patients. There was also evidence of this phenotype predominating in geographic regions such as South America, Asia, and in European countries such as Spain and Portugal, according to the Transthyretin Amyloidosis Outcomes Survey (THAOS) [26]. In a study from Spain, the neurological phenotype was mainly associated with the variant form, similar to the results of this study [27].

The cardiological phenotype was found in 53.9% of the patients in the registry. In patients with ATTRwt, there was a predominance of the cardiological phenotype (89.9%) compared to ATTRv (43.9%). Notably, the incidence of the cardiological phenotype in ATTRv was much greater than that reported in the Italian registry (13.5%) [1]. It is possible that this difference is due to the greater presence of the Val142Ile mutation in our registry than the Italian registry, in which cardiological impairment is predominant [6, 7]. On the other hand, it is also plausible to assume a selection bias of patients with a cardiological phenotype in our registry since most of the centers involved were centers specializing in cardiology.

Cardiac amyloidosis is a progressive disease that is one of the etiologies of HF and requires costs and resources for the follow-up and treatment of patients. According to the NYHA classification, most patients were classified as CF II, which is consistent with previous studies [27, 28].

In the past, there was no standardized therapeutic approach for heart failure and no specific treatment for the disease. With the positive results of multicenter studies on medications that stabilize transthyretin protein, improvements in quality of life and survival were demonstrated in this population. In our study, diuretics were the most commonly used medications. TTR stabilizing therapy (tafamidis) was the most frequently reported among the specific treatments for this disease, although it was used by a very low, because unfortunately, in Brazil, the use of tafamidis for patients with cardiac amyloidosis was not approved by government agencies.

One of the red flags is the low voltage of the QRS complex on the ECG and the increased thickness of the ventricular wall on the echocardiogram, which raises the suspicion of amyloid cardiomyopathy [29]. Another suggestive sign on the ECG is apical longitudinal strain sparing, which was present in 31.3% of the patients in this study. This finding suggests the use of an easily recognizable and reproducible method to distinguish ATTR-CM from other causes of LV hypertrophy, as described in the study by Phelan et al. [30]. Given the relatively high sensitivity and specificity of these imaging findings compared to those of other echocardiographic parameters, the British Society of Echocardiography Guideline recommends that all patients with increased LV wall thickness undergo strain imaging, not just those patients with suspected cardiac amyloidosis. This approach encourages suspicion of and facilitates the diagnosis of these entities [31].

On the other hand, after excluding AL amyloidosis, positive cardiac scintigraphy is also suggestive of ATTR-CM, the 1-h image being more sensitive and the 3-h image being more specific for the diagnosis [32]. The heart/contralateral uptake ratio (H/CL) is calculated as the ratio of the mean count from the heart region of interest to the mean count from the contralateral chest. An H/CL ratio > 1.5 on a 1-h image and an H/CL > 1.3 on a 3-h image are highly suggestive of ATTR-CM [33].

In this study, CMR showed increased T1 map and ECV values, in addition to subendocardial late gadolinium enhancement, which is characteristic of patients with ATTR-CM and is also in agreement with a previous study [27].

### Study limitations

While our study is the largest report of patients with TTR amyloidosis to date in Brazil we only access patients in state of São Paulo, the biggest state in Brazil with 44 million inhabitants.

We excluded some patients because they did not have genetic data to classify them as ATTRv or ATTRwt. Another limitation was that examinations were not done in all patients and was a limitation of defining phenotypes. Some patients, although young and asymptomatic, underwent genetic screening due to a positive family history of a known case, hence other complementary tests were not conducted. However, we analyzed the data when they were available at the participating centers. A significant portion of subjects did not have adequate follow-up for inclusion in survival analysis.

## Conclusion

The REACT registry is the first Brazilian epidemiological registry, based on collaboration among the referral centers for cardiac amyloidosis in the state of São Paulo. We detected a higher proportion of ATTRv form, with predominance of Val50Met and Val142Ile mutations, reflecting the Portuguese and afrodescendant ethnic composition of Brazilian population. Neurological and cardiological phenotypes are the initial presentation of disease in half of the patients. The diagnosis is made in most cases by a cardiologist and neurologist. The use of complementary methods can increase the awareness of the disease, allowing an early diagnosis and specific treatment. Tafamidis was the specific drug terapy most often used in this population, despite a low number of patients.

A greater understanding and awareness of the disease will allow specific measures for early diagnosis and specific treatment and will help the physician to improve the quality of life and mortality in patients with amyloidosis in Brazil.

## Data Availability

The datasets generated during the current study are available in the Mendeley Data repository, https://data.mendeley.com/datasets/dx4z7zx639/1

## Abbreviations

ATTR: Transthyretin amyloidosis
ATTRv: Variant form transthyretin amyloidosis
ATTRwt: Wild-type transthyretin amyloidosis
TTR: Transthyretin
CM: Cardiomyopathy
PN: Polyneuropathy
HF: Heart failure
REDCap: Research Electronic Data Capture
CKD: Chronic kidney disease
BMI: Body mass index
NYHA-FC: New York Heart Association functional class
^99m^Tc-PYP: ^99M^Technetium-pyrophosphate
ECG: Electrocardiography
CMR: Cardiac magnetic resonance
AF: Atrial fibrillation/flutter
HRm: Mean heart rate
LVEF: Left ventricular ejection fraction
IVS: Interventricular septal thickness
PWT: Posterior wall thickness
LA: Left atrium
LVDD: Left ventricular diastolic diameter
LVSD: Left ventricular systolic diameter
RVDD: Right ventricle diastolic diameter
AS: Apical sparing
ECV: Extracellular volume
LGE: Late gadolinium enhancement
IQR: Interquartile range
χ2: Chi-Square test
H/CL: Heart/contralateral
